# Detection of Electrophysiological Activity of Amygdala during Anesthesia Using Stereo-EEG: A Preliminary Research in Anesthetized Epileptic Patients

**DOI:** 10.1155/2020/6932035

**Published:** 2020-10-08

**Authors:** Tao Liang, Fan Wu, Yongxing Sun, Baoguo Wang

**Affiliations:** ^1^Department of Anesthesiology, Sanbo Brain Hospital, Capital Medical University, Beijing, China; ^2^Department of Anesthesiology, Affiliated Hospital of Inner Mongolia Medical University, Inner Mongolia, China

## Abstract

Recent studies of anesthesia mechanisms have focused on neuronal network and functional connectivity. The stereo-electroencephalography (SEEG) recordings provide appropriate temporal and spatial resolution to study whole-brain dynamics; however, the feasibility to detect subcortical signals during anesthesia still needs to be studied with clinical evidence. Here, we focus on the amygdala to investigate if SEEG can be used to detect cortical and subcortical electrophysiological activity in anesthetized epileptic patients. Therefore, we present direct evidence in humans that SEEG indeed can be used to record cortical and subcortical electrophysiological activity during anesthesia. The study was carried out in propofol-anesthetized five epileptic patients. The electrophysiology activity of the amygdala and other cortical areas from anesthesia to the recovery of consciousness was investigated using stereo-EEG (SEEG). Results indicated that with the decrease of propofol concentration, power spectral density (PSD) in the delta band of the amygdala significantly decreased. When it was close to recovery, the correlation between the amygdala and ipsilateral temporal lobe significantly decreased followed by a considerable increase when awake. The findings of the current study suggest SEEG as an effective tool for providing direct evidence of the anesthesia mechanism.

## 1. Introduction

Recent studies of anesthesia mechanisms have focused on neuronal network and functional connectivity [1, 2]. Anesthetic agents comprise a wide variety of molecules acting on numerous receptors, channels, and other protein targets in the body. At a small dose, anesthetics first suppress thinking, focused attention, and working memory. As the dose is increased, consciousness and voluntary responsiveness begin to fade. Functional neuroimaging by now has become a principal tool to study the neural correlates of consciousness [3]. In most studies, functional magnetic resonance imaging (fMRI) and electroencephalography (EEG) have been used to build neuronal networks [4, 5]. However, direct observation of the brain deeper structure activity with EEG or fMRI measures is difficult [6, 7]. Stereo-EEG (SEEG) is used to determine the localization of the epileptic focus before surgery in pharmaco-resistant epileptic patients [8]. Under the guidance of navigation, the electrode is implanted into the brain to directly record the electrical activity of the area. There are multiple contacts on the electrode; thus, they can be used for recording the cortical and subcortical electrophysiological activity accurately [9]. This precise process needs to be finished under general anesthesia. Therefore, SEEG provides us with a powerful tool to study neuronal brain dynamics with high temporal and good spatial resolution [10].

Being a less invasive and effective alternative investigative tool, SEEG is used for recording the seizures with a three-dimensional analysis of the epileptic zone [11]. It has been widely used for the localization of the epileptic zones in different types of epilepsies. SEEG electrodes are generally preferred over ECoG grids when the lateralization of the seizures is unknown or is expected to be in deeper brain structures, such as the insula or hippocampus [12]. A recent published systemic analysis has shown that SEEG efficiently identified epileptic zones in about 92% of the patients who underwent SEEG before surgery [13]. Similarly, SEEG has been used effectively and successfully for the investigation of various types of epilepsies including temporal lobe epilepsy, extratemporal epilepsy, insular epilepsy, and various other types as summarized in a recently published review [14]. SEEG provides sparser coverage spanning more, bilateral brain regions including deeper structures. SEEG avoids unnecessary craniotomies and the associated morbidity, hospital stay, and costs [12].

Despite its wider implications in epileptic investigations, the feasibility of SEEG for the detection of subcortical signals during anesthesia still needs to be studied with clinical evidence. In this work, with the help of SEEG, we describe the electrophysiology activity of the amygdala and other cortical areas from anesthesia to recovery of consciousness (ROC) in epileptic patients and try to verify the feasibility of SEEG as an anesthesia research method.

## 2. Materials and Methods

### 2.1. Participants

This study was approved by the Ethics committee of Sanbo Brain Hospital, Capital Medical University (SBNK-YJYS-2019-009-01), and was registered in the Chinese Clinical Trial Registry (registration number: ChiCTR2000029067). All participants provided their written informed consent before the experiments. The whole experimental process was carried out according to the Declaration of Helsinki.

### 2.2. Inclusion and Exclusion Criteria

#### 2.2.1. Inclusion Criteria

Following inclusion criteria was followed by the inclusion of the patients in this study: (1) patients with intractable epilepsy and who needed SEEG electrode implantation, (2) both male and female patients in 18-40-year age range, (3) grade I or II patients as classified by the American Society of Anesthesiologists (ASA) without serious systemic diseases (such as heart, lung, liver, and renal failure), and (4) patients capable of signing informed consent.

#### 2.2.2. Exclusion Criteria

The following criteria as used for excluding the patients from the study: (1) patients with a history of anesthesia within six months; (2) breastfeeding or pregnant female patients; (3) patients participate in other clinical trials in the last four weeks; (4) patients using sleeping pills and analgesics for a long time or alcoholic patients; (5) extremely anxious, panicked patients having communication difficulties.

## 3. Experimental Procedures

### 3.1. Experimental Design and Procedure

**Figure 1:**
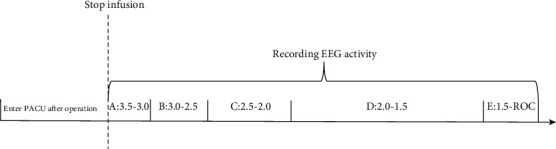
Schematic representation of the experimental procedure.

In this study, the electrophysiological activity of the amygdala was observed from anesthesia to recovery. Total intravenous anesthesia (TIVA) was used for all patients. Propofol was administered as a target-controlled infusion (TCI) (3.5 *μ*g/mL of plasma) based on the pharmacokinetic model by Marsh et al. [15]. When all electrodes had been implanted, patients were transported to the Post Anesthesia Care Unit (PACU). SEEG activity from anesthesia to recovery was recorded in PACU. The following time periods were marked accurately: (A) propofol 3.5-3.0 *μ*g/mL, (B) propofol 3.0-2.5 *μ*g/mL, (C) propofol 2.5-2.0 μg/mL, (D) propofol 2.0-1.5 μg/mL, and (E) 1.5 μg/mL-ROC. The experiment procedure is shown in Figure 1. The average period was 101 s, 123 s, 244 s, 644 s, and 185 s for investigating A, B, C, D, and E, respectively, as shown in Table S5.

### 3.2. EEG Recordings

Electrodes were implanted in five patients included in the study. The total number of electrodes for each patient was as follows: yqy =15; wl =10; zyk =15; zlx =11; and wy =13. Multiple electrodes were implanted in each patient for observing the occurrence of epilepsy. However, we only focused on the amygdala and ipsilateral temporal lobe. Exact locations of the implanted electrodes are given in Table S1 of the Supplementary data file. SEEG activity was recorded from platinum-iridium semiflexible multicontact intracerebral electrodes, with a maximum of 16 contacts per electrode (Huake Hengsheng, China). The implantation of electrodes was guided by the navigation machine which was up to 0.2 mm. The basic parameters of these implants are given in Figure S1 and Table S2 of the Supplementary data file. Each electrode contained different sites which are shown in Table S3. The distance between the recording sites was 1.5 mm. As mentioned earlier, we only focused on the amygdala and ipsilateral temporal lobe; thus, many channels were excluded as shown in Table S4, and the details are given in the supplementary data file. EEG signals were collected using the Nicolet One EEG-64 device (Nicolet Corp., USA) with a sampling frequency of 1024 Hz. The signals were band-passed at 1–55 Hz to avoid baseline drift and high-frequency noise. Signals were excluded from the analysis of those contacts that were located in (i) white matter (as assessed by MRI), (ii) epileptogenic zone (as confirmed by postsurgical assessment), (iii) over regions of documented alterations of the cortical tissue, and (iv) exhibited spontaneously or evoked epileptiform activity during recovery from anesthesia. The data were imported into Matlab (2016b) in ASCII format for further analysis.

### 3.3. EEG Analysis

Data analysis was performed with Brainstorm [16], which is documented and freely available for download online under the GNU general public license (http://neuroimage.usc.edu/brainstorm).

#### 3.3.1. Power Spectrum Analysis

This process is aimed at evaluating the power of EEG signals at different frequencies, using Welch's method. Signals were split in overlapping windows of a given length, and the Fourier Transform (FFT) of each of these short segments and the average power of the FFT coefficients were calculated for all the overlapping windows [17]. The window length used for the splitting of the signal was 10 seconds, and the window overlap ratio was 50%. The result was grouped in frequency bands: delta 2~4 Hz, theta 5~7 Hz, alpha 8~12 Hz, and beta 15~29 Hz.

#### 3.3.2. Time-Frequency Analysis

In this study, the wavelet transform was used to decompose EEG data to finish time-frequency analysis and to observe the changes of amygdala EEG in the process of anesthesia recovery. The purpose of this procedure was to observe the changes of different frequencies in the time domain during anesthesia to recovery. Time-frequency decomposition was computed based on the convolution of the signal with a series of complex Morlet wavelets. Wavelets had a variable resolution in time and frequency, while designing the wavelet, a trade-off between temporal and spectral resolution was decided. Therefore, in this study, the central frequency of 1 Hz and time resolution (FWHM) of 3 s were set [18]. The results were presented by the power of the wavelet coefficients.

#### 3.3.3. EEG Correlation Analysis

The correlation is the basic approach to show the dependence or association among two random EEG signals [19]. The implantation plan of five patients involved both amygdala and the ipsilateral temporal lobe. Therefore, the correlation of the amygdala and temporal lobe in different periods was calculated in this study.

## 4. Statistical Analysis

Statistical analysis was performed using Matlab (2016b). The power spectral data from different electrodes of the amygdala were analyzed by one-way ANOVA followed by Tukey's honestly significant difference post hoc test. A paired sample *t*-test was applied to analyze the correlation at different times. A *p* value of < 0.05 was considered statistically significant.

## 5. Result

### 5.1. General Information

Five epilepsy Asian patients (three men and two women), aged in 19-31 years (mean of 24.6 years) and weighing 68-90 kg (mean of 78.4 kg), were enrolled. The time for operation was 1.5-3 h (mean of 2.2 h), time for recovery of consciousness was 20-37 min (mean of 26.8 min), and fluid of crystal and colloid were 1940 mL and 600 mL, respectively. The ranges for fluid of crystal and colloid were 1200~2700 mL and 500~1000 mL, respectively. All of the locations of electrodes are shown in Table 1. Multiple electrodes were implanted in each patient for observing the occurrence of epilepsy. However, we only focused on the amygdala and ipsilateral temporal lobe in this study. The illustration of the method of implant surgery is shown in Figure S2. The representative locations of the implanted electrodes are shown in Figure S3. For the correlation of the amygdala and temporal lobe, the middle temporal gyrus of the temporal lobe was investigated. In the case of the amygdala, its location was determined according to the postoperative imaging data and was considered as a whole.

### 5.2. Changes in the Overall PSD of the Amygdala

**Figure 2:**
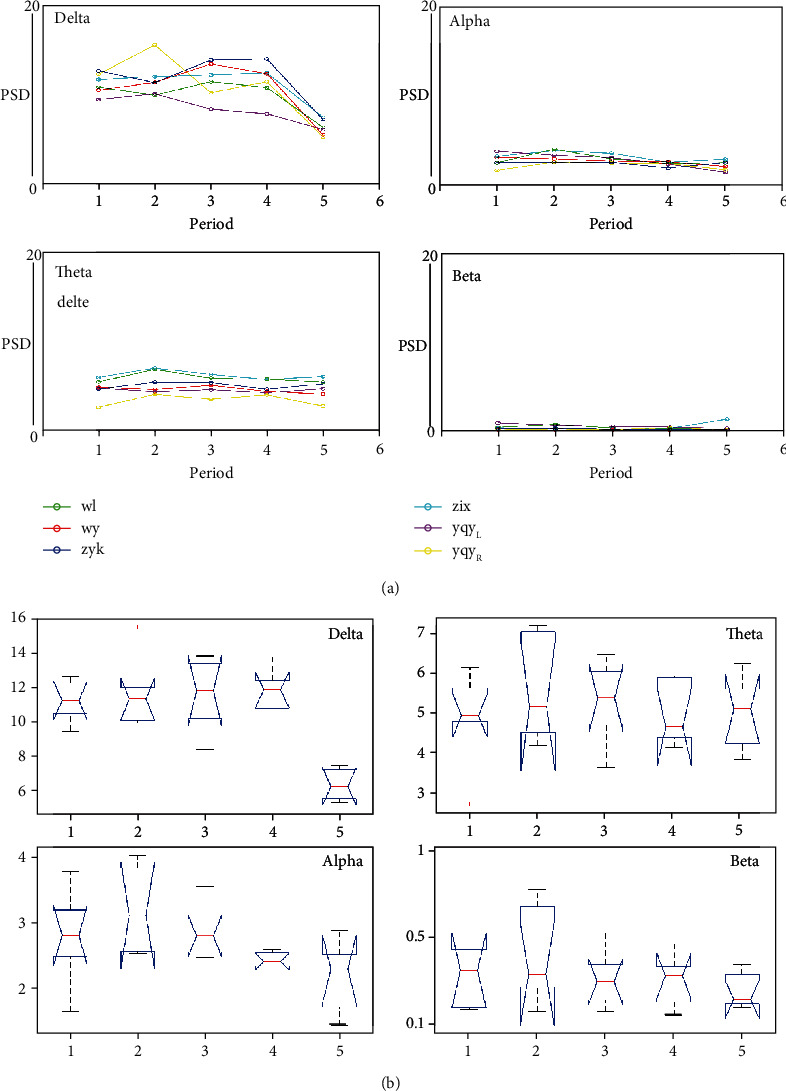
Evaluation of the PSD of the amygdala in the frequency domain where (a) delta band is the larger proportion in the whole process, and (b) there is no significant difference among theta, alpha, and beta bands.

We evaluated the PSD of the amygdala in the frequency domain, and then, from the perspective of the time domain, we tried to demonstrate the correlation between them. To investigate the PSD of amygdale from anesthesia to recovery, EEG powers at each frequency band were calculated. It was found that when anesthesia was close to recovery, the PSD of the delta band decreased (Figure 2). One-way ANOVA analysis showed significant difference at delta band (*F* = 10.83 and *p* < 0.001), but not at theta (*F* = 0.42 and *p* = 0.792), alpha (*F* = 3.36 and *p* = 0.124), and beta (*F* = 0.84 and *p* = 0.513) bands (Table S6).

### 5.3. Time-Frequency Analysis

**Figure 3:**
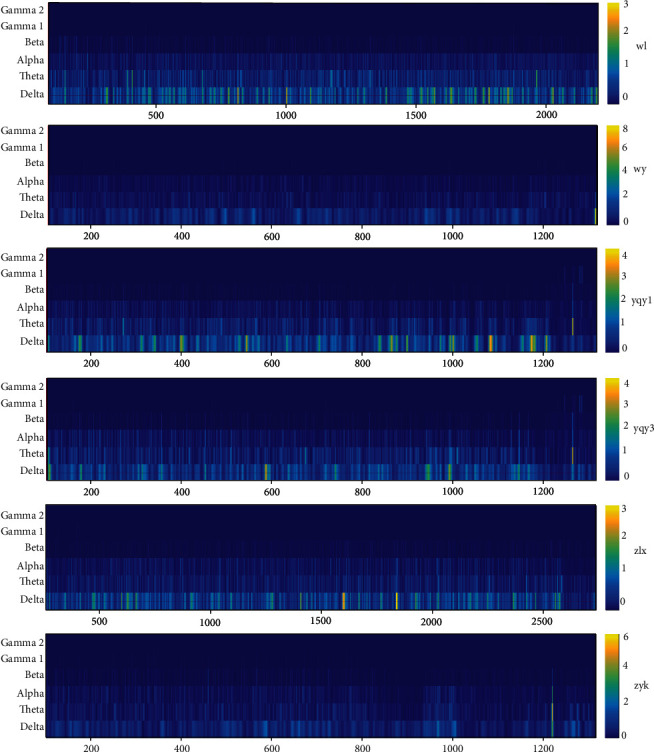
Time-frequency of amygdale indicating reproducibility of the method.

Time-frequency analysis was conducted to demonstrate any change in the amygdala during the stable anesthesia. In the time-frequency analysis, it can be seen that all of the data show some similarity in the whole process. With the decrease of propofol concentration in plasma, the power of the delta band was always the highest. The order was delta>theta>alpha>beta. We found that this method can be well repeated (Figure 3).

### 5.4. Correlation of Amygdala and Temporal Lobe

**Figure 4:**
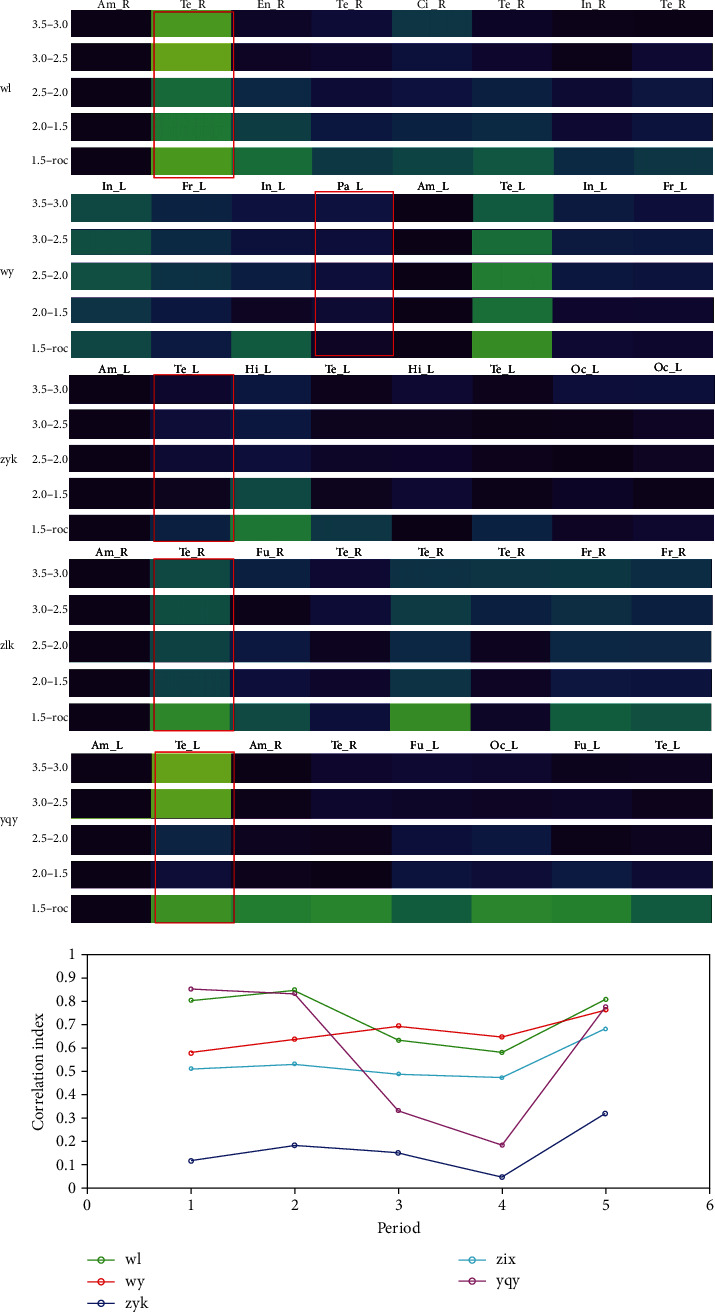
Correlation of amygdala and temporal lobe.

Next, we sought to investigate the status of the correlation between the amygdala and the ipsilateral temporal lobe under anesthesia. The extent of correlation changes of different propofol concentrations is presented in Figure 4. A paired *t-*test was used to analyze the changes in the correlation among different periods. The following effects were observed: at first, the correlation was relatively high in the following time periods: (A) propofol, l3.5-3.0 *μ*g/mL; (B) propofol, 3.0-2.5 *μ*g/mL; and (C) propofol, 2.5-2.0 *μ*g/mL. When the concentration of propofol decreased to (D) 2.0-1.5 *μ*g/mL, a significant decline was observed. However, a significant increase as observed when the drug concentration further decreased as shown in Table S7.

## 6. Discussion

The main aim of the current study is to identify the feasibility of using SEEG to observe subcortical electrophysiological activities from anesthesia to recovery of consciousness directly. We observed that low-frequency activity (delta) of the amygdala was strong under anesthesia and decreased gradually when it was close to awake. A previous study showed that propofol-induced anesthesia reduces the episodic memory reconsolidation in humans [20]. We also found that the correlation between the amygdala and the ipsilateral temporal lobe was stable under anesthesia. When the propofol concentration was decreased to 2.0-1.5 *μ*g/mL, a significant decrease in the correlation was observed. Previous studies also suggest the suppression of the complexity of regions sparsely connected with large-scale brain networks as a mechanism of propofol-induced alteration in oriented reactivity to stimulation [21]. When the drug concentration further decreased, a significant increase was observed. This can be related to the regulation of fear under light anesthesia [22–24]. Effective connectivity, its dynamics, and its directionality are altered by propofol [25].

The amygdala is an important part of the subcortical limbic system [26]. Previous studies used PET and fMRI to measure the amygdala activity during anesthesia [27, 28]. However, these studies did not measure the spatial resolution in real time. In contrast, we used SEEG to record EEG data to locate the target area in real time [29]. Moreover, the previous studies give a very localized information about SEEG [30], whereas we try to give a more deep insight into the SEEG activity of the amygdala during anesthesia. Previously published reports of electrophysiological changes of the amygdala were studies for Spontaneous Inflammatory Pain-Associated Activation of Pain Networks [31], change throughout postnatal development [32], and emotion categories of complex visual stimuli [33]. However, these studies did not evaluate the electrophysiological changes of the amygdala during anesthesia in epileptic patients. Time-frequency analysis of this study also observed that there was no significant change in the amygdala of the five patients during the stable anesthesia.

In some studies, EEG was used to observe the changes in the brain under anesthesia [34, 35]. The spatial resolution of EEG is not precise enough. Moreover, fMRI is also used to indirectly measure the activity of neurons before and after anesthesia in some studies. The spatial resolution of this method is good; however, it cannot monitor dynamically in real time. Previously Li et al. [36] suggest the use of the Laplacian re-reference for preprocessing in studies of broadband gamma and low-frequency oscillatory activity in SEEG signals. This assists in the emerging and unique SEEG method for the exploration of neural dynamics across the entire human brain; however, it was not specific for targeted location. In this study, we use SEEG to record EEG data, which can accurately locate the target area under the guidance of the navigation robot and meet the time and space resolution simultaneously. However, this is an invasive operation that can only be used for patients with intractable epilepsy. All of the five patients involved the amygdala and the ipsilateral temporal lobe in this study. Therefore, in the choice of research method, we observe the PSD of the amygdala in the frequency domain firstly, and then, from the perspective of the time domain, we tried to observe the correlation between them. This method has been widely used in electrophysiology, but it is not the best technique for finding the connectivity matrices. Multiple studies have reported high rates of seizure freedom following SEEG localization to the temporal lobe, ranging from 72.7% to 80% [9, 37]. However, it still can provide valuable information when we deal with a few narrow-banded signals in the time domain. Finally, we observed the changes of the amygdala from anesthesia to recovery with time-frequency analysis in different patients.

Recently, many researchers have tried to use SEEG to study the brain from different perspectives [38, 39]. However, the application of SEEG in the research of the anesthesia mechanism is still rare. Besides, the feasibility of this method used in the research of the anesthesia mechanism needs to be further demonstrated. Therefore, we provide direct evidence for the repeatability in this study.

## 7. Limitation

First, the sample size of this study is limited. In future studies, we will enlarge the sample size to cover more brain areas. Secondly, this study mainly focuses on propofol, and for other drugs, further research is needed. Moreover, this is an exploratory research. The main purpose is to verify the feasibility of this method. Additionally, in the current study, resting oscillations may be quite abnormal in these patients and will need to design a strategy to overcome the abnormal oscillations. A large cohort study is still needed to further clarify the potential mechanisms of anesthesia.

## 8. Conclusion

Electrophysiology activity of the amygdala and other cortical areas from anesthesia to the recovery of consciousness was investigated in propofol-anesthetized five epileptic patients using stereo-EEG (SEEG). With the decrease of propofol concentration, PSD in the delta band of the amygdala decreased significantly. When it was close to awakening, the correlation between the amygdala and ipsilateral temporal lobe significantly decreased followed by a considerable increase when awake. Therefore, SEEG can be used to provide direct evidence for the study of the anesthesia mechanism.

## Figures and Tables

**Table 1:** Basic information of electrodes locations (^∗^In: insular lobe; Fr: frontal lobe; Pa: parietal lobe; Am: amygdale; Te: temporal lobe; Fu: fusiform gyrus; Oc: occipital lobe; Ci: cingulate gyrus; En: entorhinal cortex; Hi: hippocampus).

ID	Sex	Age (years)	Height (cm)	Weight (kg)	Surgery time (h)	ROC (min)	Crystal (mL)	Colloid (mL)	Electrode location							
									1	2	3	4	5	6	7	8
zlx	Male	19	175	75	1.5 h	37	2100	500	Am_R	Te_R	Fu_R	Te_R	Te_R	Te_R	Fr_R	Fr_R
zyk	Male	22	171	80	2 h	20	2100	500	Am_L	Te_L	Hi_L	Te_L	Hi_L	Te_L	Oc_L	Oc _L
wy	Female	31	176	68	2 h	22	2700	500	In_L	Fr_L	In_L	Pa_L	Am_L	Te_L	In_L	Fr_L
yqy	Male	23	185	90	2.5 h	25	1600	1000	Am_L	Te_L	Am_R	Te_R	Fu _L	Oc _L	Fu_L	Te_L
wl	Female	28	155	79	3 h	30	1200	500	Am_R	Te_R	En_R	Te_R	Ci _R	Te_R	In_R	Te_R

## Data Availability

There is no such data.

## Supplementary Materials

Supplementary Materials In the supplementary materials, we present the following contents: Table S1, Table S2, Table S3, Table S4, Table S5, Table S6, Table S7, Figure S1, Figure S2, Figure S3, and the method of implant surgery.
