# Supplementary material for: Detection of Electrophysiological Activity of Amygdala during Anesthesia Using Stereo-EEG: A Preliminary Research in Anesthetized Epileptic Patients
Source: Biomed Res Int. 2020 Oct 8;2020:6932035. doi: 10.1155/2020/6932035 (PMC7568817; doi:10.1155/2020/6932035)
Supplement: Supplementary Materials — In the supplementary materials, we present the following contents: Table S1, Table S2, Table S3, Table S4, Table S5, Table S6, Table S7, Figure S1, Figure S2, Figure S3, and the method of implant surgery. [file 6932035.f1.docx]

**Detection of electrophysiological activity of amygdala during anesthesia using stereo-EEG: A preliminary research in anesthetized epileptic patients**

Tao Liang^1^, Fan Wu^2^, Yongxing Sun^1^, Baoguo Wang^1^*

1. Department of Anesthesiology, Sanbo Brain Hospital, Capital Medical University, Beijing, China
2.Department of Anesthesiology, Affiliated Hospital of Inner Mongolia Medical University, Inner Mongolia, China

Tao Liang: [ltmzvip@163.com](mailto:ltmzvip@163.com)

Fan Wu: [wfmzvip@163.com](mailto:wfmzvip@163.com)

Yongxing Sun: [8793705@163.com](mailto:8793705@163.com)

Baoguo Wang: [wbgsbnk@163.com](mailto:wbgsbnk@163.com)

**Table S1:** Location of implanted electrodes in the amygdala and ipsilateral temporal lobe (from inside to outside)

| **Patient** | **Electrode location** |
| --- | --- |
| **yqy** | Left amygdala to left temporal lobe/ right amygdala to right temporal lobe; |
| **wl** | Right amygdala to right temporal lobe; |
| **zyk** | Left amygdala to left temporal lobe; |
| **zlx** | Right amygdala to right temporal lobe; |
| **wy** | Left amygdala to left temporal lobe. |


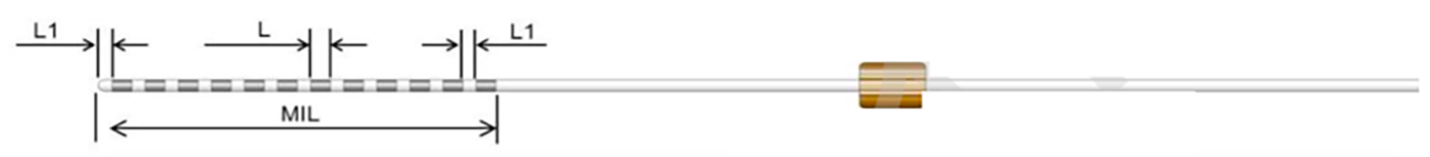


**Figure S1:** The example of the electrode used

**Table S2:** Basic parameters of the implant used

| Contacts Number | Diameter of Contact | Length of Contact/L | Interval distance of Contacts/L1 | Effective Length/MIL | Length of Electrode |
| --- | --- | --- | --- | --- | --- |
| 12 | 0.8 mm | 2 mm | 1.5 mm | 42 mm | 400~500 mm |

**Table S3:** Number of contact sites on each electrode

| Patient | Number of electrode contact sites | |
| --- | --- | --- |
|  | **Right amygdala** | **Left amygdala** |
| yqy | 16 | 16 |
| wl | 12 | ----------- |
| zyk | ----------- | 16 |
| zlx | 16 | -------------- |
| wy | --------- | 16 |

**Table S4:** Number of excluded electrodes

| **Patient** | **Number of excluded electrodes** |
| --- | --- |
| **yqy** | 13 electrodes were excluded; |
| **wl** | 9 electrodes were excluded; |
| **zyk** | 14 electrodes were excluded; |
| **zlx** | 10 electrodes were excluded; |
| **wy** | 12 electrodes were excluded. |

**Table S5:** Time periods and their average for each investigation

| Patient | A | B | C | D | E |
| --- | --- | --- | --- | --- | --- |
| Yqy | 127s | 150s | 347s | 628s | 126s |
| Wl | 96s | 141s | 311s | 940s | 190s |
| Zyk | 72s | 117s | 194s | 540s | 221s |
| Zlx | 90s | 101s | 190s | 562s | 250s |
| W y | 120s | 109s | 180s | 550s | 141s |
| Average | 101s | 123s | 244s | 644s | 185s |

**Table S6:** PSD of six amygdale electrodes at various bands

| Frequency | Time period | | | | | F | p-value |
| --- | --- | --- | --- | --- | --- | --- | --- |
|  | **A3.5-3.0** | **B3.0-2.5** | **C2.5-2.0** | **D2.0-1.5** | **E1.5-ROC** |  |  |
| δ | 11.25 ±1.19 | 11.73 ±2.04 | 11.60 ±2.05 | 11.48 ±2.07 | 6.34 ±0.88 | 10.83 | 3.11*10^-5^* |
| θ | 4.85 ±1.17 | 5.54 ±1.30 | 5.27 ±1.01 | 4.94 ±0.78 | 5.01 ±0.89 | 0.42 | 0.792 |
| α | 2.79 ±0.73 | 3.20 ±0.63 | 2.87 ±0.39 | 2.37 ±0.24 | 2.19 ±0.54 | 3.36 | 0.124 |
| β | 0.40 ±0.30 | 0.42 ±0.25 | 0.30 ±0.15 | 0.29 ±0.13 | 0.23 ±0.09 | 0.84 | 0.513 |

*p=3.11*10^-5^<0.001 statistically significant

**Table S7:** Comparison of correlation between the amygdala and temporal lobe

| ID | Correlation | | | | | | | |
| --- | --- | --- | --- | --- | --- | --- | --- | --- |
|  | **A** | **B** | | **C** | | **D** | | **E** |
| Wl | 0.802 | 0.848 | | 0.632 | | 0.679(0.579) | | 0.807 |
| Wy | 0.576 | 0.637 | | 0.694 | | 0.646 | | 0.764 |
| Zyk | 0.115 | 0.180 | | 0.148 | | 0.043 | | 0.317 |
| Zlx | 0.510 | 0.531 | | 0.488 | | 0.471 | | 0.479(0.679) |
| Yqy | 0.851 | 0.831 | | 0.329 | | 0.180 | | 0.775 |
| Pair-t test | A vs B:0.092 | | B vs C:0.211 | | C vs D:0.033* | | D vs E:0.025* | |

**Method of implant surgery**

1. **Anesthesia process:**

Dosages of anesthetics were determined according to standard dosing requirements. The intravenous induction drugs were sufentanyl (2–4 μg/kg), rocuronium (0.1 mg/kg). Propofol was administered as a target-controlled infusion (TCI) (3.5ug/mL of plasma) based on the pharmacokinetic model by Marsh et al. Recovery of consciousness (ROC) time point was determined by response to verbal commands. Recordings were obtained in the PACU room.

1. **Surgery process:**

Before surgery, electrophysiologists planned for implantation according to the patient's symptoms and MRI data. And the data were inputted into the navigation machine. During surgery, neurosurgeon implanted electrodes under the guidance of the navigation machine. After all of the electrodes were implanted, patients had a CT scan. Then imaging experts fused CT and MRI data to verify the position of electrodes. In this study, we chose electrode contact with the help of imaging experts.


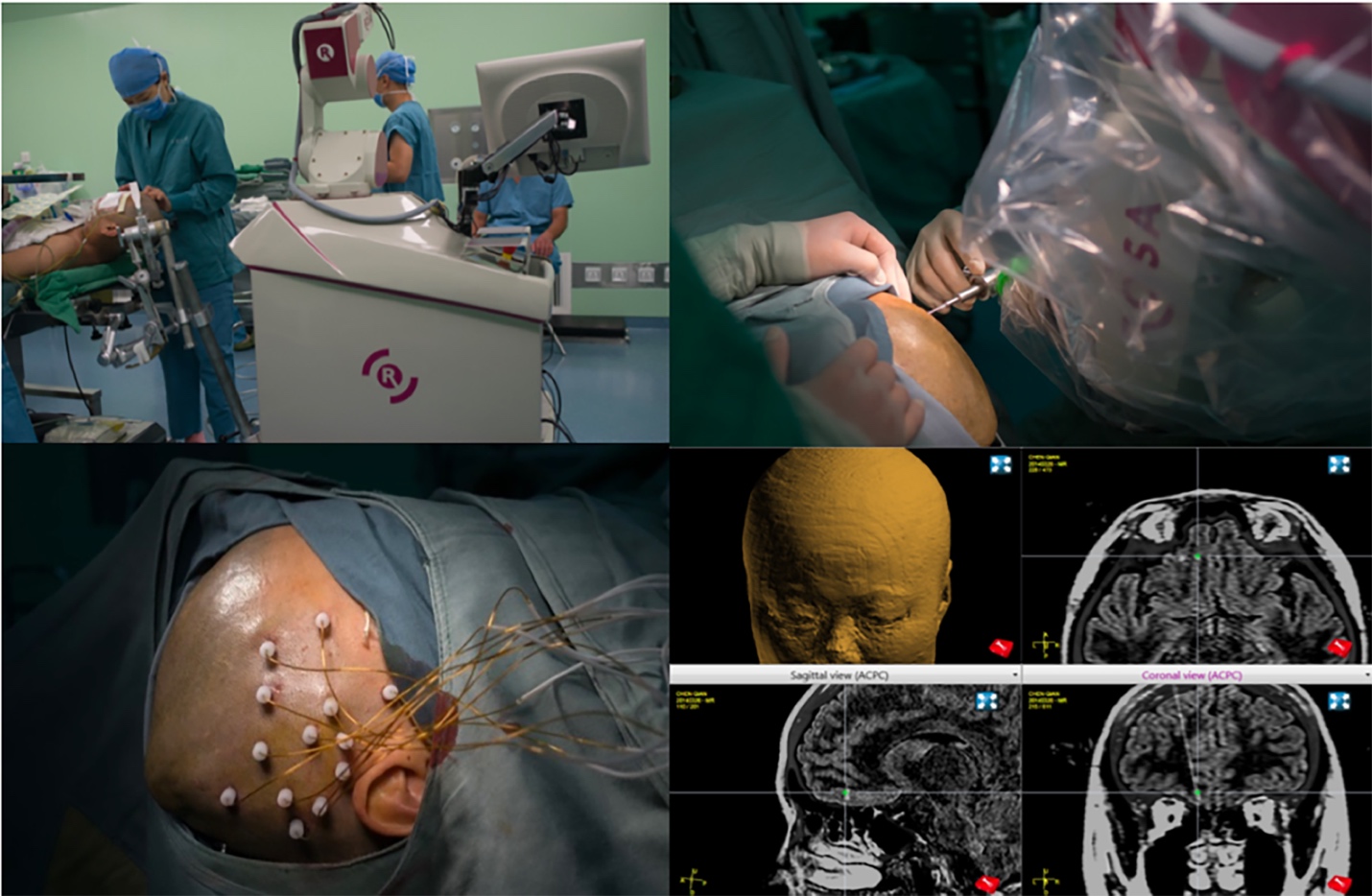


**Figure S2:** Illustration of the method of implant surgery


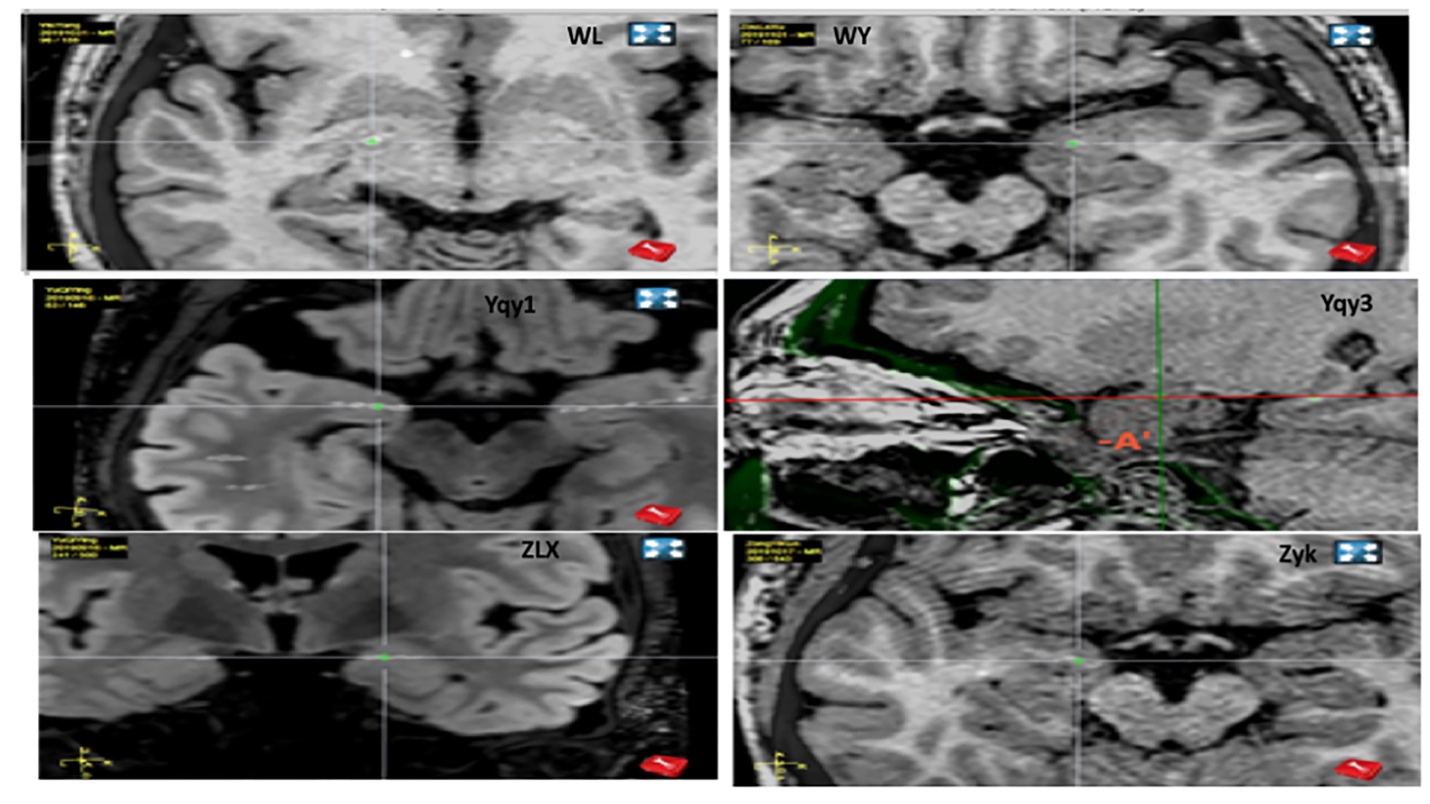


**Figure S3:** Representative MRI scans for the positions of the implanted electrode. Patient identifiers are shown in each imag of patient scan.
